# Supplementary material for: Subicular neurons encode concave and convex geometries
Source: Nature. 2024 Mar 6;627(8005):821–9. doi: 10.1038/s41586-024-07139-z (PMC10972755; doi:10.1038/s41586-024-07139-z)
Supplement: Supplementary file 1 — Reporting Summary [file 41586_2024_7139_MOESM1_ESM.pdf]

Corresponding author(s): Yanjun Sun and Lisa Giocomo

Last updated by author(s): Jan 8, 2024

Reporting Summary

Nature Portfolio wishes to improve the reproducibility of the work that we publish. This form provides structure for consistency and transparency in reporting. For further information on Nature Portfolio policies, see our Editorial Policies and the Editorial Policy Checklist.

Statistics

For all statistical analyses, confirm that the following items are present in the figure legend, table legend, main text, or Methods section.

n/a	Confirmed	
☐	☒	The exact sample size (*n*) for each experimental group/condition, given as a discrete number and unit of measurement
☐	☒	A statement on whether measurements were taken from distinct samples or whether the same sample was measured repeatedly
☐	☒	The statistical test(s) used AND whether they are one- or two-sided<br>*Only common tests should be described solely by name; describe more complex techniques in the Methods section.*
☐	☒	A description of all covariates tested
☐	☒	A description of any assumptions or corrections, such as tests of normality and adjustment for multiple comparisons
☐	☒	A full description of the statistical parameters including central tendency (e.g. means) or other basic estimates (e.g. regression coefficient) AND variation (e.g. standard deviation) or associated estimates of uncertainty (e.g. confidence intervals)
☐	☒	For null hypothesis testing, the test statistic (e.g. *F*, *t*, *r*) with confidence intervals, effect sizes, degrees of freedom and *P* value noted<br>*Give P values as exact values whenever suitable.*
☐	☒	For Bayesian analysis, information on the choice of priors and Markov chain Monte Carlo settings
☒	☐	For hierarchical and complex designs, identification of the appropriate level for tests and full reporting of outcomes
☐	☒	Estimates of effect sizes (e.g. Cohen's *d*, Pearson's *r*), indicating how they were calculated

Our web collection on statistics for biologists contains articles on many of the points above.

Software and code

Policy information about availability of computer code

Data collection	Calcium Imaging data were acquired using custom-developed and validated software (Compiled DAQ Software OLD version 1.0.0, https://github.com/daharoni/Miniscope_DAQ_Software)
Data analysis	MATLAB packages that were used for the manuscript include NoRMCorre v1.0.0 (https://github.com/flatironinstitute/NoRMCorre) for image motion correction, image-registration v1.0.0 (github.com/fordanic/image-registration) for image alignment across days, CNMF-E v1.1.2 (https://github.com/zhoupc/CNMF_E) for Ca++ signal extraction, and OASIS v1.0.0 (https://github.com/zhoupc/OASIS_matlab) for Calcium signal deconvolution. MATLAB 2020a and and GraphPad Prism 9 were used to perform statistical analyses. Calcium imaging data were further preprocessed and analyzed using MATLAB based packages and scripts (https://github.com/yanjuns/Sun_et_al_2024_Nature).

For manuscripts utilizing custom algorithms or software that are central to the research but not yet described in published literature, software must be made available to editors and reviewers. We strongly encourage code deposition in a community repository (e.g. GitHub). See the Nature Portfolio guidelines for submitting code & software for further information.

Data

Policy information about availability of data

All manuscripts must include a data availability statement. This statement should provide the following information, where applicable:

- Accession codes, unique identifiers, or web links for publicly available datasets
- A description of any restrictions on data availability
- For clinical datasets or third party data, please ensure that the statement adheres to our policy

Calcium imaging data generated in this study are available on Mendeley Data: https://doi.org/10.17632/5sj8d5vtg2.1
Calcium imaging data were further preprocessed and analyzed using MATLAB based packages and scripts: https://github.com/yanjuns/Sun_et_al_2024_Nature

Research involving human participants, their data, or biological material

Policy information about studies with human participants or human data. See also policy information about sex, gender (identity/presentation), and sexual orientation and race, ethnicity and racism.

Reporting on sex and gender	NA
Reporting on race, ethnicity, or other socially relevant groupings	NA
Population characteristics	NA
Recruitment	NA
Ethics oversight	NA

Note that full information on the approval of the study protocol must also be provided in the manuscript.

Field-specific reporting

Please select the one below that is the best fit for your research. If you are not sure, read the appropriate sections before making your selection.

☒ Life sciences ☐ Behavioural & social sciences ☐ Ecological, evolutionary & environmental sciences

For a reference copy of the document with all sections, see nature.com/documents/nr-reporting-summary-flat.pdf

Life sciences study design

All studies must disclose on these points even when the disclosure is negative.

Sample size	Sample sizes were based upon on convention in the field (Sun et al., 2022; Mallory et al., 2021; Low et al., 2021; Campbell et al., 2021; Alexander et al., 2020; Hardcastle et al., 2015). The number of mice used in each set of experiments ranged from 7 to 10.
Data exclusions	After a certain period post-surgery, the imaging quality began to decline in some animals, and this thus led to slight variations in the number of mice used in each set of experiments, ranging from 7 to 10. We evaluated the imaging quality for each mouse before executing each set of experiments. No mice were excluded from the analyses as long as the experiments were executed. For experiments with two identical sessions for a given condition (e.g., Fig. 1 and 2), sessions with less than 3 identified corner cells were excluded to minimize measurement noise in spike rates. This criterion only resulted in the exclusion of one session from one mouse in Fig. 2e.
Replication	Up to six mice were used as a cohort for each batch of experiments. All experiments were repeated with at least two different cohorts of mice and the results are reproducible.
Randomization	Randomization is not applicable to this study, as all animals in a given experiment were subjected to the same behavioral and imaging protocols.
Blinding	Blinding is not applicable to this study, as there were no separated groups in the experiments.

Reporting for specific materials, systems and methods

We require information from authors about some types of materials, experimental systems and methods used in many studies. Here, indicate whether each material, system or method listed is relevant to your study. If you are not sure if a list item applies to your research, read the appropriate section before selecting a response.

Materials & experimental systems

n/a	Involved in the study
☒	☐ Antibodies
☒	☐ Eukaryotic cell lines
☒	☐ Palaeontology and archaeology
☐	☒ Animals and other organisms
☒	☐ Clinical data
☒	☐ Dual use research of concern
☒	☐ Plants

Methods

n/a	Involved in the study
☒	☐ ChIP-seq
☒	☐ Flow cytometry
☒	☐ MRI-based neuroimaging

Animals and other research organisms

Policy information about studies involving animals; ARRIVE guidelines recommended for reporting animal research, and Sex and Gender in Research

Laboratory animals	For subiculum imaging, 8 Camk2a-Cre; Ai163 mice (Ref 43, 4 male and 4 female), 1 Camk2-Cre mouse (female, JAX: 005359), and 1 C57BL/6 mouse (male) were used. For CA1 imaging, 12 Ai94; Camk2a-tTA; Camk2a-Cre (JAX id: 024115 and 005359) mice (7 male and 5 female) were used. Mice were group housed with same-sex littermates until the time of surgery. At the time of surgery, mice were 8 -12 weeks old. After surgery mice were singly housed at 21 -22°C and 29 - 41% humidity. Mice were kept on a 12-hour light/dark cycle and had ad libitum access to food and water in their home cages at all times.
Wild animals	No wild animals were used in this study
Reporting on sex	Data from both males and females were combined for analysis, as we did not observe sex differences in, for example, corner cell proportions, spike rates to different corners angles, or concavity and convexity.
Field-collected samples	No field collected samples were used in this study.
Ethics oversight	All procedures were approved by the Institutional Animal Care and Use Committee at Stanford University School of Medicine and the University of California, Irvine.

Note that full information on the approval of the study protocol must also be provided in the manuscript.

Plants

Seed stocks	NA
Novel plant genotypes	NA
Authentication	NA
